# Heart Girth as a Predictor of Body Weight in Lactating Cows

**DOI:** 10.3390/ani16060938

**Published:** 2026-03-17

**Authors:** Silvia Magro, Alberto Guerra, Pietro Sartor, Massimo De Marchi, Mauro Penasa

**Affiliations:** Department of Agronomy, Food, Natural Resources, Animals and Environment, University of Padova, Viale dell’Università 16, 35020 Legnaro, PD, Italy; alberto.guerra@unipd.it (A.G.); massimo.demarchi@unipd.it (M.D.M.); mauro.penasa@unipd.it (M.P.)

**Keywords:** Holstein, live weight, regression, Rendena, Simmental

## Abstract

Body weight (BW) is an important trait in dairy cows; however, large-scale direct measurements are challenging. Heart girth (HG) has been proposed as a practical indicator of BW, but limited information is available for lactating cows, especially for locally adapted breeds. This study aimed to develop equations to estimate BW from HG in lactating Holstein, Simmental, and Rendena cows. A total of 293 cows (94 Holstein, 52 Simmental, and 147 Rendena) were selected from 6 farms equipped with an automatic milking system located in northern Italy. Both HG and BW were recorded on the same day, with HG measured using a tape and BW using a scale integrated into the automatic milking system. For each breed, linear, quadratic, and cubic regressions of BW on HG were tested, adjusting for days in milk and parity effects. The coefficient of determination and the root mean square error were reported. The best predictive performance was obtained with models adjusted for both days in milk and parity, with the highest accuracy achieved for Holstein and Simmental cows. These results corroborate that HG is a reliable predictor of BW in lactating cows of these breeds.

## Simple Summary

Body weight is an important characteristic of dairy cows, with practical relevance for research and on-farm management. However, large-scale direct measurements are often difficult to collect under commercial farming conditions. Information on simple methods for estimating body weight in lactating cows is still limited, especially for locally adapted breeds. This study aimed to evaluate the use of heart girth as an indicator of body weight in different dairy cow breeds. The study involved lactating Holstein, Simmental, and Rendena breeds reared on farms in northern Italy. Body weight and heart girth were measured on the same day, and their relationship was evaluated using linear and nonlinear regression models. A strong association between heart girth and body weight was observed across all breeds, indicating that heart girth can be used as a reliable indicator of body weight in lactating dairy cows.

## 1. Introduction

The body weight (BW) of a dairy cow is a relevant morphological trait with practical applications in both research and farm management [1]. Body weight plays a crucial role in assessing the health status of dairy cows, and evaluating feeding and production efficiency. Monitoring BW is a valuable tool for farmers to observe the physiological and behavioral changes in dairy cows over time [1]. In addition, BW is widely used as a predictor of other important variables, including dry matter intake [2], water intake [3], urine excretion [4], methane emissions [5], and nitrogen balance [6].

The direct measurement of BW using an electronic weighing scale is difficult to implement at the population level because of the need for animal handling and the high cost of the equipment [7]. In recent years, several studies have investigated the efficiency of precision livestock farming technologies involving 3D digital imaging [8,9,10]. However, owing to their high costs, these methods are not yet applicable in all contexts, particularly on small- to medium-sized farms. Other studies have explored the possibility of estimating BW through body measurements, an approach considered one of the most traditional and widely used for predicting the BW of dairy cows [11,12,13,14]. Several morphological traits have been shown to correlate with BW, such as wither height [15], hip width [12], and body length [16]. These measurements are generally non-invasive, easy to collect on-farm, and provide useful information for BW estimation. The prediction of BW from heart girth (HG), i.e., the circumference around the thorax at the level of the heart, has been shown to be more accurate than predictions based on other morphometric measurements [17]. Developing a prediction equation based on a single body measurement such as HG would reduce sampling time and consequently facilitate large-scale phenotype collection.

To accurately evaluate BW in dairy cows, it is important to consider additional factors such as stage of lactation and parity. Indeed, cows tend to lose BW during the first two months after calving [18] due to the mobilization of body reserves to meet the nutrient demands of milk production [19], followed by a gradual recovery in the later stages. Regarding the effect of parity, heifers typically enter lactation at approximately 80% of their mature BW [20], and during their first lactation, they continue to allocate nutrients toward ongoing body development. Consequently, primiparous cows are generally lighter than multiparous cows [21].

Several studies have explored the estimation of BW using body measurements in cattle such as HG, wither height, hip width, and body length [11,12,13]. However, most of these studies focused on growing animals or specific breeds, and there is still limited research on the development of prediction equations for BW in lactating cows [14]. Moreover, there is a lack of studies targeting less widespread and locally adapted breeds. Given the marked differences in body conformation among breeds, developing breed-specific BW prediction equations is essential. In particular, specialized dairy breeds (e.g., Holstein) exhibit morphological characteristics that differ substantially from those of dual-purpose breeds, such as Simmental and Rendena, and this may affect the accuracy of generalized prediction models. Holstein is the predominant dairy breed in Italy, followed by Simmental, and Rendena, an autochthonous breed of northeastern Italy [22].

This study aimed to develop equations to predict BW from HG in primiparous and multiparous Holstein, Simmental, and Rendena cows of different lactation stages.

## 2. Materials and Methods

### 2.1. Data Collection

**Figure 1:**
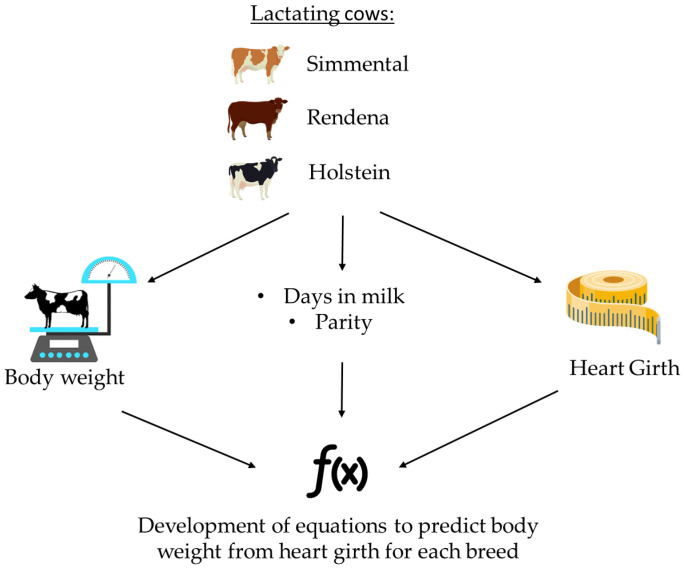
Experimental design.

Between February and July 2024, 293 lactating cows (94 Holstein, 52 Simmental, and 147 Rendena) were selected from 6 farms located in the Veneto region of northern Italy. All farms were equipped with automatic milking systems integrated with weighing scales. For each cow, HG and BW were recorded on the same day, with HG measured using a weight tape, and BW calculated as the average of three measurements from the weighing scale of the automatic milking system. Days in milk (DIM) and parity were also recorded (Figure 1).

### 2.2. Statistical Analysis

The analysis was conducted using R software v. 4.4.2 [23]. For each breed, descriptive statistics were calculated. Differences in BW and HG between breeds were assessed using a one-way analysis of variance, followed by post hoc pairwise comparisons, to evaluate the need for breed-specific prediction equations.

Prediction models were developed using linear regression model with the ‘trainControl’ function available in the package ‘Caret’ of the R software v. 4.4.2, as described by Kuhn [24]. The models were fine-tuned using 10-fold cross-validation repeated 5 times. For each breed, linear, quadratic, and cubic regressions of BW on HG were tested. In addition, the equations were adjusted for DIM and parity according to the following combinations: HG alone, HG + DIM, HG + parity, and HG + DIM + parity. For each equation, outliers were identified as observations with residuals exceeding ± 2.5 standard deviations (SD) from the mean residual [25]. The fitting statistics included the coefficient of determination (R^2^), the root mean square error (RMSE), and the mean absolute error (MAE).

## 3. Results

### 3.1. Mean and Variation

**Figure 2:**
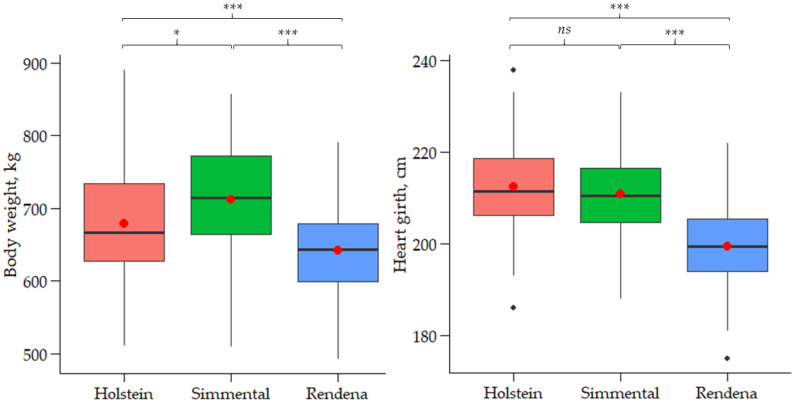
Distribution of body weight measured using the weighing scale of the automatic milking system and heart girth measured using a tape in Holstein, Simmental, and Rendena breeds. Red dots represent the mean; the lower and upper edges of the boxes represent the first and third quartiles, respectively; the midlines within the boxes indicate the median. The statistical significance is indicated as *** *p* < 0.001; * *p* < 0.05; *ns*, not significant.

The means and SD of HG, BW, DIM, and parity for each breed are reported in Table 1. The distribution of BW measured using the weighing scale integrated into the automatic milking system, as well as that of HG, is depicted in Figure 2, along with the significance of the post hoc pairwise comparisons.

On average, Holstein and Simmental had wider HG (212.40 cm and 210.90 cm, respectively) than Rendena cows (199.50 cm), with the difference being statistically significant (*p* < 0.001). Simmental cows were heavier (712.20 kg) than Holstein (678.70 kg; *p* < 0.05) and Rendena (642.10 kg; *p* < 0.001). The average DIM was 145.60, 171.00, and 170.80 days for Holstein, Simmental, and Rendena cows, respectively, and parity averaged 1.99, 2.37, and 2.39. Within breeds, BW had greater variability than HG. Specifically, coefficient of variation (CV) of BW ranged from 9.92% (Rendena) to 11.19% (Holstein), whereas CV of HG ranged from 4.14% (Holstein) to 4.89% (Simmental). Much larger variability was observed for parity and days in milk, with CV from 61.81% (Holstein) to 71.31% (Simmental) for the former and 44.60% (Simmental) to 75.04% (Holstein) for the latter. Differences in BW and HG highlight the importance of developing breed-specific prediction equations to estimate BW from HG.

### 3.2. Prediction of Body Weight from Heart Girth Within Breed

The regression coefficients and predictive performance statistics for the estimation of BW from HG, adjusted for DIM and/or parity, are reported in Table 2, Table 3 and Table 4 for the Holstein, Simmental, and Rendena breeds, respectively. Each table shows the results of four sets of predictors: HG alone, HG + DIM, HG + parity, and HG + DIM + parity. Linear, quadratic, and cubic equations of HG were fitted for each predictor set. The percentage of outliers across the different equations varied among breeds: ≤ 5.5% for Holstein (Table 2), ≤ 5.8% for Simmental (Table 3), and ≤ 8.8% for Rendena (Table 4).

Considering the performance of HG alone, all three models (linear, quadratic, and cubic) showed similar results for the Holstein breed, with the best performance obtained from the linear equation (R^2^ = 0.75; RMSE = 35.13 kg; Table 2). In contrast, for the Simmental breed, the cubic model showed the best performance (R^2^ = 0.72; RMSE = 37.31 kg; Table 3). For the Rendena breed, lower R^2^ values were observed, with similar performance across the three types of equations (R^2^ = 0.58; Table 4).

In Holsteins, the inclusion of DIM in the prediction equations did not substantially improve the model performance. Conversely, incorporating parity into the model improved performance, with R^2^ ranging from 0.78 for linear and quadratic equations to 0.81 for the cubic equation, RMSE ≤ 31.75 kg, and MAE ≤ 25.96 kg. The best performance was achieved when the model was adjusted for both DIM and parity using a cubic equation, resulting in an R^2^ of 0.86, RMSE of 26.96 kg, and MAE of 20.57 kg (Table 2).

For the Simmental breed, model performance did not improve with the inclusion of DIM alone but showed enhancement when parity was added, both individually and in combination with DIM. The highest predictive accuracy was obtained by adjusting the cubic model for both DIM and parity, achieving an R^2^ of 0.83, RMSE of 29.86 kg, and MAE of 23.60 kg (Table 3).

For the Rendena breed, the prediction equations improved with the inclusion of DIM in the model, with R^2^ ranging from 0.60 for the cubic equation to 0.64 for the quadratic equation, RMSE ≤ 37.89 kg, and MAE ≤ 32.00 kg. When only parity was included, the models achieved R^2^ values ≤ 0.61. The best performance was obtained using a linear equation adjusted for DIM and parity (R^2^ = 0.65, RMSE = 37.83 kg, and MAE = 31.98 kg; Table 4).

## 4. Discussion

### 4.1. Descriptive Statistics

Although the prediction of BW from body measurements has been previously studied for the Holstein breed, both in heifers and cows [26,27,28], similar analyses for the Simmental and Rendena breeds are lacking. Indeed, while Holstein is the most widespread breed in Italy, with 1,147,858 cows monitored for milk production and quality [22], Simmental and Rendena are less represented, and have received comparatively less attention in studies on BW prediction. These dual-purpose breeds account for significantly fewer heads in Italy than Holstein under milk production and quality control (60,645 and 3428 cows, respectively) [22]. In particular, the Rendena breed is mainly located in the Trentino-Alto Adige and Veneto regions [22].

The BW of Holstein cows observed in the present study was higher than that reported by Finocchiaro et al. [29] on 3256 Italian Holsteins. The lower BW in the study of Finocchiaro et al. [29] is attributable to the fact that only first-parity cows were considered, whereas our study included both primiparous and multiparous cows. The BW and body measurements observed in the present study were in line with those reported by Gruber et al. [14], who observed BW from 400 to 1088 kg in Fleckvieh, Holstein, and Brown Swiss breeds. The same cows considered by Gruber et al. [14] had HG measurements ranging from 166 to 257 cm, although their analysis pooled different breeds together instead of evaluating them separately.

### 4.2. Prediction of Body Weight from Heart Girth

Regarding the equation to predict BW from HG, Yan et al. [30] obtained results similar to those of the present study by analyzing 146 Holstein cows with an average BW of 574 ± 74.40 kg. Specifically, the prediction model based on HG alone yielded an R^2^ of 0.78 and an RMSE of 36.30 kg. However, the model performance improved when both HG and belly girth were included, reaching an R^2^ of 0.90 and an RMSE of 23.20 kg [30]. Heinrichs et al. [17] demonstrated that HG in Holstein heifers is one of the body measurements most strongly related to BW, with R^2^ exceeding 0.90. Furthermore, Dingwell et al. [31] identified a strong association (R^2^ = 0.94) between HG and BW in 311 Holstein heifers. In contrast, Gruber et al. [14] considered 6306 cows from three breeds (Holstein, Fleckvieh, and Brown Swiss) in Austria during both the lactation and dry periods with average BW of 702 ± 88.9 kg and 788 ± 93.6 kg, respectively. Two models were developed for each period by adding body measurements to the HG. The first model included belly girth combined with HG and yielded an R^2^ of 0.83 and an RMSE of 37.00 kg during lactation and an R^2^ of 0.80 and an RMSE of 41.30 kg during the dry period. The second model was developed by adding both belly girth and hip width to HG, resulting in an R^2^ of 0.84 and an RMSE of 36.50 kg during lactation and an R^2^ of 0.80 and an RMSE of 39.90 kg during the dry period. However, the prediction equations developed by Gruber et al. [14] considered all breeds together. The breed-related differences in BW and HG observed in the present study (Figure 2) highlighted the need to develop breed-specific prediction equations for estimating BW from the HG. Moreover, our results demonstrated that adjusting the equations for DIM enhanced the model performance (Table 2, Table 3 and Table 4). Indeed, cows tend to lose BW in the first two months after calving [18] due to the mobilization of body reserves to satisfy the nutrient demands for milk production [19], with subsequent weight recovery in the later stages of lactation when milk production decreases. This high and rapid energy mobilization may depress the cows’ metabolic and reproductive systems [32]. In general, a greater decline in BW is observed in cows that produce more milk at the beginning of lactation [33]. Adjusting the model for parity also improved the predictive performance (Table 2, Table 3 and Table 4). This effect was more pronounced in Simmental and Holstein than in Rendena breed. This improvement may be related to the fact that primiparous cows have not yet reached their full mature BW, which contributes to greater variability in BW compared to multiparous cows, whose BW tends to be more stable [20].

Prediction models performed worse for Rendena compared to Holstein and Simmental, likely because, as an autochthonous breed, it shows a different body conformation that HG alone cannot fully capture. Consequently, including additional body measurements could contribute to improving the accuracy of the prediction models for Rendena.

## 5. Conclusions

The breed-specific differences in BW and HG observed in this study underscore the importance of developing tailored prediction equations for estimating BW from HG in Holstein, Simmental, and Rendena cows. The regression models showed a strong predictive performance for BW based on HG across all breeds, particularly Holstein and Simmental. In general, adjusting the models for DIM and parity further improved the predictive performance of these equations. Future research should increase the sample size to check for potential improvement in model accuracy and validate the equations using an external dataset.

## Figures and Tables

**Table 1:** Descriptive statistics ^1^ of heart girth, body weight, days in milk, and parity for Holstein, Simmental, and Rendena breeds.

Item	Mean	SD	CV, %
Holstein (*n* = 94)			
Heart girth, cm	212.40	8.80	4.14
Body weight, kg	678.70	75.94	11.19
Days in milk	145.60	109.26	75.04
Parity	1.99	1.23	61.81
Simmental (*n* = 52)			
Heart girth, cm	210.90	10.31	4.89
Body weight, kg	712.20	78.05	10.96
Days in milk	171.00	76.27	44.60
Parity	2.37	1.69	71.31
Rendena (*n* = 147)			
Heart girth, cm	199.50	8.47	4.25
Body weight, kg	642.10	63.67	9.92
Days in milk	170.80	123.00	72.01
Parity	2.39	1.51	63.18

^1^ SD = standard deviation; CV = coefficient of variation.

**Table 2:** Regression coefficients ^1^ of heart girth (HG), days in milk (DIM), and parity, and predictive performance statistics ^2^ of the equations for body weight of Holstein cows.

	Equation	Intercept	HG1	HG2	HG3	DIM	Parity	Outliers, %	R^2^	RMSE	MAE
HG	Linear	−720.14 ***	6.595 ***					4.4	0.75	35.13	29.21
	Quadratic	−266.42	2.286 *	0.01 *				5.5	0.75	37.40	30.84
	Cubic	30,260.00 ^†^	−436.40 ^†^	2.108 ^†^	−0.00334 ^†^			4.4	0.74	35.36	29.67
HG + DIM	Linear	−608.60 ***	5.995 ***			0.08526 *		5.5	0.74	35.87	30.09
	Quadratic	2162.86	−20.894 *	0.065 ^†^		0.08958 *		5.5	0.75	35.14	29.36
	Cubic	29,680.00	−427.60	2.065 ^†^	−0.00327 ^†^	0.08709 *		5.5	0.72	36.67	30.85
HG + Parity	Linear	−552.84 ***	5.674 ***				14.72 ***	4.4	0.78	31.06	25.33
	Quadratic	−1379.00	13.50 *	−0.019 *			14.74 ***	4.4	0.78	31.75	25.96
	Cubic	20,430.00	−296.80	1.449 ^†^	−0.00231 ^†^		14.13 ***	4.4	0.81	30.51	24.47
HG + DIM + Parity	Linear	−436.67 ***	5.021 ***			0.10787 **	15.54 ***	5.5	0.78	31.66	26.02
	Quadratic	−1588.00	15.92 *	−0.026 *		0.10840 **	15.52 ***	5.5	0.77	32.20	26.62
	Cubic	23,940.00	−345.70	1.676	−0.00266 ^†^	0.09534 **	14.14 ***	4.4	0.86	26.96	20.57

^1^ *** *p* < 0.001; ** *p* < 0.01; * *p* < 0.05; ^†^ *p* < 0.10. ^2^ R^2^, coefficient of determination; RMSE, root mean square error; MAE, mean absolute error.

**Table 3:** Regression coefficients ^1^ of heart girth (HG), days in milk (DIM), and parity, and predictive performance statistics ^2^ of the equations for body weight of Simmental cows.

	Equation	Intercept	HG1	HG2	HG3	DIM	Parity	Outliers %	R^2^	RMSE	MAE
HG	Linear	−358.384 *	5.147 ***					5.8	0.63	45.07	36.53
	Quadratic	−10,570.00 ***	102.00 ***	−0.2291 ***				3.8	0.71	40.99	35.31
	Cubic	83,950.00 **	−1256.00 **	6.256 **	−0.01031 **			3.8	0.72	37.31	31.66
HG + DIM	Linear	−365.341 *	5.167 ***			0.01611		3.8	0.59	46.17	37.61
	Quadratic	−10,590.00 ***	102.30 ***	−0.2299 ***		−0.03121		3.8	0.71	41.80	35.70
	Cubic	82,870.00 **	−1240.00 **	6.184 **	−0.01019 ***	−0.02898		3.8	0.73	39.06	33.03
HG + Parity	Linear	−360.752 *	5.205 ***				−4.1279	3.8	0.58	45.84	37.99
	Quadratic	−10,110.00 ***	97.71 ***	−0.2191 ***			0.4245	1.9	0.69	43.09	37.05
	Cubic	83,870.00 *	−1254.00 **	6.251 **	−0.01030 **		0.04409	5.8	0.79	31.98	28.19
HG + DIM + Parity	Linear	−361.20 ^†^	5.206 ***			0.00101	−4.12	5.8	0.70	39.18	35.29
	Quadratic	−9718.00 ***	94.06 ***	−0.2107 ***		−0.04191	0.92050	5.8	0.71	38.52	35.77
	Cubic	83,100.00 *	−1243.00 **	6.199 **	−0.01022 **	−0.02938	−0.1459	5.8	0.83	29.86	23.60

^1^ *** *p* < 0.001; ** *p* < 0.01; * *p* < 0.05; ^†^ *p* < 0.10. ^2^ R^2^, coefficient of determination; RMSE, root mean square error; MAE, mean absolute error.

**Table 4:** Regression coefficients ^1^ of heart girth (HG), days in milk (DIM), and parity, and predictive performance statistics ^2^ of the equations for body weight of Rendena cows.

	Equation	Intercept	HG1	HG2	HG3	DIM	Parity	Outliers, %	R^2^	RMSE	MAE
HG	Linear	−436.32 ***	5.4349 ***					6.8	0.58	38.71	33.59
	Quadratic	1863.37	−17.6865	0.058				6.1	0.58	39.25	33.83
	Cubic	−17,650.00	278.50	−1.438	0.002515 *			6.1	0.58	39.01	33.58
HG + DIM	Linear	−429.32 ***	5.4883 ***			−0.1082 ***		8.8	0.63	36.35	30.93
	Quadratic	891.03	−7.81671	0.03344		−0.10339 ***		8.8	0.64	36.25	30.87
	Cubic	−18,440.00	284.40	−1.437	0.002461 *	−0.08908 **		7.5	0.60	37.89	32.00
HG + Parity	Linear	−412.03 ***	5.383 ***				−5.944 **	7.5	0.60	37.43	32.58
	Quadratic	2301.47 ^†^	−21.9187	−21.91865 ^†^			−6.52625 **	7.5	0.61	36.91	31.95
	Cubic	−20,250.00	320.40	−1.661	0.002907 *		−6.614 **	7.5	0.61	31.99	31.99
HG + DIM + Parity	Linear	−430.66 ***	5.54102 ***			−0.08643 **	−5.50576 **	6.8	0.65	37.83	31.98
	Quadratic	1436.77	−13.26296	0.04725		−0.07999 **	−5.87467 **	6.8	0.62	37.66	31.73
	Cubic	−13,030.00	206.10	−1.06	0.001859 *	−0.07004 *	−5.998 **	6.1	0.62	37.72	31.83

^1^ *** *p* < 0.001; ** *p* < 0.01; * *p* < 0.05; ^†^ *p* < 0.10. ^2^ R^2^, coefficient of determination; RMSE, root mean square error; MAE, mean absolute error.

## Data Availability

None of the data was deposited in an official repository. The data that support the study are available from the corresponding author upon reasonable request.

## Abbreviations

The following abbreviations are used in this manuscript:

BW	Body Weight
CV	Coefficient of Variation
R^2^	Coefficient of Determination
DIM	Days in Milk
HG	Heart Girth
MAE	Mean Absolute Error
RMSE	Root Mean Square Error
SD	Standard Deviation

## References

[1] Song X., Bokkers E.A.M., Van der Tol P.P.J., Koerkamp P.G., Van Mourik S. (2018). Automated body weight prediction of dairy cows using 3-dimensional vision. J. Dairy. Sci..

[2] Hoffman P.C., Weigel K.A., Wernberg R.M. (2008). Evaluation of equations to predict dry matter intake of dairy heifers. J. Dairy. Sci..

[3] Appuhamy J.A.D.R.N., Judy J.V., Kebreab E., Kononoff P.J. (2016). Prediction of drinking water intake by dairy cows. J. Dairy. Sci..

[4] Nennich T.D., Harrison J.H., VanWieringen L.M., St-Pierre N.R., Kincaid R.L., Wattiaux M.A., Davidson D.L., Block E. (2006). Prediction and evaluation of urine and urinary nitrogen and mineral excretion from dairy cattle. J. Dairy. Sci..

[5] Moraes L.E., Strathe A.B., Fadel J.G., Casper D.P., Kebreab E. (2014). Prediction of enteric methane emissions from cattle. Glob. Change Biol..

[6] Spanghero M., Kowalski Z.M. (2021). Updating analysis of nitrogen balance experiments in dairy cows. J. Dairy. Sci..

[7] Simanungkalit G., Hegarty R.S., Cowley F.C., McPhee M.J. (2020). Evaluation of remote monitoring units for estimating body weight and supplement intake of grazing cattle. Animal.

[8] Cotticelli A., Verde M.T., Liccardo A., Alteriis G.D., Lamonaca F., Matera R., Neglia G., Peric T., Prandi A., Bonavolontà F. (2023). On the use of 3D camera to accurately measure volume and weight of dairy cow feed. Acta IMEKO.

[9] Gebreyesus G., Milkevych V., Lassen J., Sahana G. (2023). Supervised learning techniques for dairy cattle body weight prediction from 3D digital images. Front. Genet..

[10] Hansen F., Smith M.L., Smith L.N., Jabbar K.A., Forbes D. (2018). Automated monitoring of dairy cow body condition, mobility and weight using a single 3D video capture device. Comput. Ind..

[11] Banos G., Coffey M.P. (2012). Prediction of liveweight from linear conformation traits in dairy cattle. J. Dairy. Sci..

[12] Enevoldsen C., Kristensen T. (1997). Estimation of body weight from body size measurements and body condition scores in dairy cows. J. Dairy. Sci..

[13] Martins B.M., Mendes A.L.C., Silva L.F., Moreira T.R., Costa J.H.C., Rotta P.P., Chizzotti M.L., Marcondes M.I. (2020). Estimating body weight, body condition score, and type traits in dairy cows using three dimensional cameras and manual body measurements. Livest. Sci..

[14] Gruber L., Ledinek M., Steininger F., Fuerst-Waltl B., Zottl K., Royer M., Krimberger K., Mayerhofer M., Egger-Danner C. (2018). Body weight prediction using body size measurements in Fleckvieh, Holstein, and Brown Swiss dairy cows in lactation and dry periods. Arch. Anim. Breed..

[15] Tasdemir S., Urkmez A., Inal S. (2011). Determination of body measurements on the Holstein cows using digital image analysis and estimation of live weight with regression analysis. Comput. Electron. Agric..

[16] Lukuyu M.N., Gibson J.P., Savage D.B., Duncan A.J., Mujibi F.D.N., Okeyo A.M. (2016). Use of body linear measurements to estimate liveweight of crossbred dairy cattle in smallholder farms in Kenya. SpringerPlus.

[17] Heinrichs A.J., Rogers G.W., Cooper J.B. (1992). Predicting body weight and wither height in Holstein heifers using body measurements. J. Dairy. Sci..

[18] Admina N.G., Paliy A.P., Petrov R.V., Nagorna L.V., Kovalenko L.M., Nazarenko S.M., Sevastianov V.V. (2024). Influence of growth intensity of black and white dairy cattle on their reproduction and productivity under free housing. Regul. Mech. Biosyst..

[19] Peiter M., Caixeta L., Endres M.I. (2023). Association between change in body weight during early lactation and milk production in automatic milking system herds. JDS Commun..

[20] Berry D.P., Buckley F., Dillon P. (2011). Relationship between live weight and body condition score in Irish Holstein-Friesian dairy cows. Irish J. Agr. Food Res..

[21] Ungerfeld R., Hötzel M.J., Scarsi A., Quintans G. (2011). Behavioral and physiological changes in early-weaned multiparous and primiparous beef cows. Animal.

[22] Associazione Italiana Allevatori (AIA) (2024). Bollettino Online. Statistiche Ufficiali. http://bollettino.aia.it/Contenuti.aspx?CD_GruppoStampe=RS&CD_Specie=C4.

[23] R Core Team (2025). R: A Language and Environment for Statistical Computing.

[24] Kuhn M. (2008). Building predictive models in R using the caret package. J. Stat. Softw..

[25] Tedeschi L.O. (2006). Assessment of the adequacy of mathematical models. Agric. Syst..

[26] Hurst T.S., Lopez-Villalobos N., Boerman J.P. (2021). Predictive equations for early-life indicators of future body weight in Holstein dairy heifers. J. Dairy. Sci..

[27] Heinrichs A.J., Heinrichs B.S., Jones C.M., Erickson P.S., Kalscheur K.F., Nennich T.D., Heins B.J., Cardoso F.C. (2017). Verifying Holstein heifer heart girth to body weight prediction equations. J. Dairy. Sci..

[28] Kuzuhara Y., Kawamura K., Yoshitoshi R., Tamaki T., Sugai S., Ikegami M., Kurokawa Y., Obitsu T., Okita M., Sugino T. (2015). A preliminarily study for predicting body weight and milk properties in lactating Holstein cows using a three-dimensional camera system. Comput. Electron. Agr..

[29] Finocchiaro R., van Kaam J.B.C.H.M., Marusi M., Cassandro M. Body Weight Prediction and Genetic Parameter Estimation Based on Type Traits in Italian Holstein Cows 2017. https://server01.anafi.it/Latteco/articoli/2017/Icar-Giu-2017.pdf.

[30] Yan T., Mayne C.S., Patterson D.C., Agnew R.E. (2009). Prediction of body weight and empty body composition using body size measurements in lactating dairy cows. Livest. Sci..

[31] Dingwell R.T., Wallace M.M., McLaren C.J., Leslie C.F., Leslie K.E. (2006). An evaluation of two indirect methods of estimating body weight in Holstein calves and heifers. J. Dairy Sci..

[32] Aleri J.W., Hine B.C., Pyman M.F., Mansell P.D., Wales W.J., Mallard B., Fisher A.D. (2016). Periparturient immunosuppression and strategies to improve dairy cow health during the periparturient period. Res. Vet. Sci..

[33] Buckley F., O’sullivan K., Mee J.F., Evans R.D., Dillon P. (2003). Relationships among milk yield, body condition, cow weight, and reproduction in spring-calved Holstein-Friesians. J. Dairy Sci..

